# Phytochemical Analysis of *Pinus cembra* Heartwood—UHPLC-DAD-ESI-MS^n^ with Focus on Flavonoids, Stilbenes, Bibenzyls and Improved HPLC Separation

**DOI:** 10.3390/plants12193388

**Published:** 2023-09-25

**Authors:** Fabian Alperth, Anna Schneebauer, Olaf Kunert, Franz Bucar

**Affiliations:** 1 Department of Pharmacognosy, Institute of Pharmaceutical Sciences, University of Graz, Beethovenstraße 8, 8010 Graz, Austria; fabian.alperth@uni-graz.at (F.A.); schneebauer.anna@gmail.com (A.S.); 2 Department of Pharmaceutical Chemistry, Institute of Pharmaceutical Sciences, University of Graz, Universitätsplatz 1, 8010 Graz, Austria; olaf.kunert@uni-graz.at

**Keywords:** *Pinus cembra*, heartwood, phytochemistry, flavonoids, stilbenes, bibenzyls, UHPLC-MS, HPLC-DAD, isolation, NMR

## Abstract

The heartwood of the Swiss Stone Pine, *Pinus cembra* L., has been scarcely investigated for secondary metabolites for a long period of time. Considering age and relative simplicity of heartwood investigations dating back to the 1940s to 1960s, we conducted the first investigation of *P. cembra* heartwood by HPLC, using UHPLC-DAD-ESI-MS^n^ and HPLC-DAD techniques in combination with isolation and NMR spectroscopy, with focus on stilbenes, bibenzyls and flavonoids. Analytical problems in the HPLC analysis of *Pinus* stilbenes and flavonoids on reversed stationary phases were also challenged, by comparing HPLC on pentafluorophenyl (PFP) and C18 stationary phases. Seven flavonoids (**1**, **2**, **3**, **7**, **8**, **11**, **12**), four stilbenes (**4**, **6**, **10**, **13**), two bibenzyls (**5**, **9**), three fatty acids (**14**, **16**, **17**) and one diterpenic acid (**15**) were detected in an ethanolic extract of *Pinus cembra* heartwood. HPLC comparison of reversed stationary phases in HPLC showed that the antifungal, antibacterial and chemosensitizing dihydropinosylvin monomethyl ether (**9**) and pinosylvin monomethyl ether (**10**) can be separated on PFP, but not on C18 material, when eluting with a screening gradient of 20–100% acetonitrile. Flavonoid separation showed additional benefits of combining analyses on different stationary phases, as flavonoids **7** and **8** could only be separated on one of two C18 stationary phases. Earlier phytochemical results for heartwood investigations were shown to be mostly correct, yet expandable. Substances **5** to **12** were found in alignment with these references, proving remarkable phytochemical analyses at the time. Evidence for the described presence of pinobanksin could not be found. Substances **1** to **4** and **13** have to our knowledge not yet been described for *P. cembra*.

## 1. Introduction

The Swiss Stone Pine, *Pinus cembra* L., is a prominent coniferous tree in the family Pinaceae. It is distributed in the Alps and Carpathian Mountains and has a long history of use for its wood as crafting and building material. The seeds are very nutritious and have culinary uses [1]. Essential oils and preparations produced from different *Pinus cembra* parts and liquors aromatized with fresh cones are commercially available. However, the phytochemistry of secondary metabolites in *Pinus cembra*, especially in wood samples, has been rather scarcely investigated for a long period of time. Results in literature have also never been compiled appropriately, even more so while credibly excluding results for *Pinus sibirica* Du Tour, which is often incorporated into the species as *Pinus cembra var. sibirica* or *Pinus cembra subsp. sibirica* [2,3]. Therefore, a chronological summary of phytochemical research concerning secondary metabolites in *Pinus cembra* L. is appropriate and provided by us in the following. Here, special focus is given to substance groups of stilbenes, bibenzyls and flavonoids. In earlier studies of *P. cembra* heartwood, the isolation of the stilbenes pinosylvin (PS), pinosylvin monomethyl ether (PSM), the corresponding bibenzyls dihydropinosylvin (DHPS), and dihydropinosylvin monomethyl ether (DHPSM) and the flavonoids chrysin, tectochrysin, pinocembrin, pinostrobin and pinobanksin was reported [4,5,6]. Griesbach and Santamour investigated anthocyanins in male cones of *P. cembra* by HPLC-UV, reporting cyanidin-3-glucoside, delphinidin-3-glucoside and peonidin-3-glucoside [7]. Slimestad published results for flavonoids in buds and young needles investigated by HPLC-DAD-MS, giving kaempferol-3-glucoside, isorhamnetin-3-glucoside, kaempferol-3-(6″-coumaroyl-glucoside) and kaempferol-3-(3″, 6″-di-coumaroyl-glucoside) [8]. For some years, scientific focus shifted to knotwood investigations, with the main stilbene and bibenzyl being PSM and DHPSM [9] and pinocembrin as major flavonoid [10,11]. Others reported total phenolics, flavonoids and proanthocyanidins in bark and needles [12], and group investigations with reference standards for fatty acids, resin acids, terpenes, phenolic compounds and saccharides [13], however, without identification of flavonoids, stilbenes and bibenzyls. Also, *P. cembra* softwood lignins were investigated by NMR spectroscopy [14]. HPLC techniques in the analysis of *P. cembra* wood samples have only been reintroduced in recent years. Patyra et al. investigated branch wood methanolic extracts by LC-DAD-ESI-MS^n^ to find fourteen compounds, of which six catechin derivatives and procyanidins, as well as taxifolin, a taxifolin hexoside, eriodictyol and pinobanksin can be assigned to the substance group of flavonoids. PSM was the only reported stilbene in *P. cembra* extracts [15]. Finally, Coniglio et al. detected PS, DHPS, chrysin and pinocembrin in *P. cembra* knotwood extracts by LC-ESI-MS [16].

As given here, composition of stilbenes, bibenzyls and flavonoids seems to vary between wood samples of different morphological parts. Considering these variances, we aim for the first investigation of *Pinus cembra* heartwood by HPLC, using UHPLC-DAD-ESI-MS^n^ and HPLC-DAD techniques, with focus on stilbenes, bibenzyls and flavonoids, compound classes of numerous biological and pharmacological effects. Thereby we also revisit some results from Erdtman et al. and Lindstedt et al. [4,5,6] with modern instrument-based chromatographic possibilities in combination with isolation and NMR spectroscopy. In course of this study, we also challenge major analytical problems in the HPLC analysis of *Pinus* stilbenes and flavonoids on reversed stationary phases. New findings in method development are of interest for the development of fast and reliable natural product isolation by HPLC.

## 2. Results and Discussion

### 2.1. UHPLC-DAD-ESI-MS^n^ Analysis

**Figure 1:**
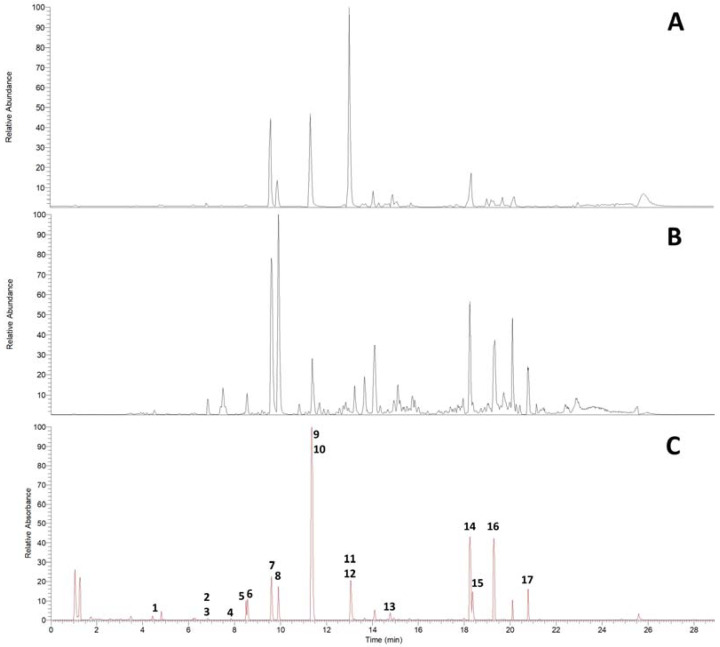
UHPLC-DAD-ESI-MS^n^ chromatograms of *Pinus cembra* heartwood ethanolic extract on RP18 stationary phase. (**A**) ESI positive mode; (**B**) ESI negative mode; (**C**) DAD-UV (Spectrum maxi-mum 190–700 nm).

**Figure 2:**
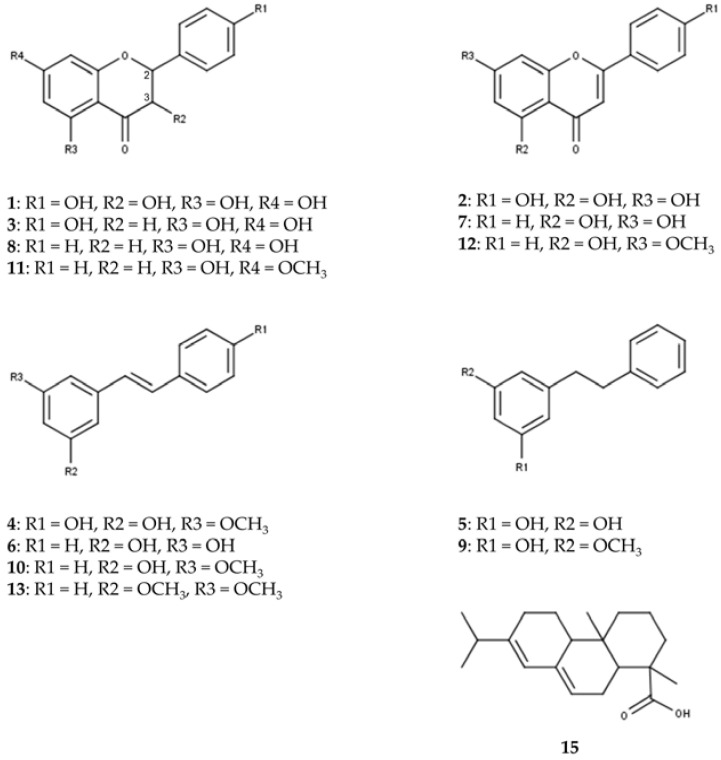
Structures of characterized substances except for common fatty acids **14**, **16** and **17**.

UHPLC on an RP18 stationary phase was employed for the initial qualitative analysis of a *Pinus cembra* heartwood ethanolic extract. Seventeen substances were characterized by interpretation of DAD-UV data and MS^n^ in positive and negative ionization mode, of which six could further be confirmed by comparison with authentic reference standards and four by subsequent isolation (2.2), as given in Table 1. A chromatogram of MS in positive and negative ionization mode and DAD-UV (190–700 nm) can be found in Figure 1; Figure 2 gives structures of all characterized substances except for common fatty acids **14**, **16** and **17**.

The constituents can be grouped into seven flavonoids (**1**, **2**, **3**, **7**, **8**, **11**, **12**), four stilbenes (**4**, **6**, **10**, **13**), two bibenzyls (**5**, **9**), three fatty acids (**14**, **16**, **17**) and one diterpenic acid (**15**). Additional data for MS^n^ in positive and negative ionization mode are given in Supplementary Materials (Tables S1 and S2).

To our knowledge, trace flavonoids **1** (aromadendrin), **2** (apigenin) and **3** (naringenin) are described for *Pinus cembra* for the first time. Previous literature mentions the occurrence of **1** and **2** in *Pinus sibirica* [17]. For flavonoid **1**, comparison with MS data in literature supports the characterization of aromadendrin rather than eriodictyol [18,19,20]. Evidence for the presence of pinobanksin could not be identified, as MS data for flavonoid **3** rather suggest the detection of naringenin [15,19,20,21,22,23]. For flavonoid **1**, indicative losses of H_2_O (18 amu) and CO (28 amu) are detected in positive ionization mode, to form [M+H-H_2_O]^+^ (*m*/*z* 271), [M+H-H_2_O-CO]^+^ (*m*/*z* 243) and [M+H-H_2_O-2CO]^+^ (*m*/*z* 215). C-ring cleavage leads to the detection of an ^1,3^A^+^-fragment (*m*/*z* 153); also, the loss of the B-ring can be recognized as [M+H-B-ring]^+^ (*m*/*z* 195). Besides several losses of small groups in negative ionization mode, the ^1,3^A^−^-fragment (*m*/*z* 151) is also detected. Flavonoid **2** shows the combined loss of H_2_O and CO in positive ionization mode to give [M+H-H_2_O-CO]^+^ (*m*/*z* 225), as well as the ^1,3^A^+^-fragment (*m*/*z* 153). The ^1,3^A^−^-fragment (*m*/*z* 151) is again detected in negative ionization mode. In positive ionization mode, the fragmentation of flavonoid **3** shows the ^1,3^A^+^-fragment (*m*/*z* 153) and ^0,4^B^+^-fragment (*m*/*z* 147) at high intensities after C-ring cleavage. In negative ionization mode, the ^1,3^A^−^-fragment (*m*/*z* 151) is detected as the most abundant fragment ion in MS^2^. Flavonoids **7** (chrysin), **8** (pinocembrin), **11** (pinostrobin) and **12** (tectochrysin) were found in accordance with older references for *Pinus cembra* [4,5,6,10,11,16].

Stilbenes **6** (pinosylvin, PS) and **10** (pinosylvin monomethyl ether, PSM) as well as corresponding bibenzyls **5** (dihydropinosylvin, DHPS) and **9** (dihydropinosylvin monomethyl ether, DHPSM) were previously described for *Pinus cembra* heartwood [4,5,6,9,15,16]. Stilbenes **4** (pinostilbene) and **13** (pinosylvin dimethyl ether) were only found in other *Pinus* species, including old references of occurrence in *Pinus sibirica* [24,25]. ESI-MS in positive ionization mode proved to give more spectral information for stilbenes than negative ionization. In positive ionization mode, stilbene **4** shows the loss of a phenolic ring [M+H-C_6_H_6_O]^+^ (*m*/*z* 149) as the most abundant fragment ion in MS^2^. This is followed by a fragmentation of −28 amu in MS^3^ (*m*/*z* 149 to 121), which could be interpreted as [M+H-C_6_H_6_O-CH_2_CH_2_]^+^ or [M+H-C_6_H_6_O-CO]^+^. In negative ionization mode, the loss of a methyl group (15 amu) gives the most abundant fragment ion [M-H-CH_3_]^-^ (*m*/*z* 226) in MS^2^. Stilbene **13** shares the most fragments in MS^2^ of the positive ionization mode with stilbene **10**, with the molecular ion [M+H]^+^ and the two heaviest fragment ions of **13** (*m*/*z* 241, 223 and 213, respectively) showing +14 amu, which corresponds to an additional methoxy- instead of hydroxy-group in stilbene **13**.

### 2.2. Isolation of Major Compounds

For further confirmation of UHPLC-DAD-ESI-MS^n^ results, isolation of several main compounds in *Pinus cembra* heartwood was performed. Open-column chromatography (CC) of the ethanolic extract on silica led to 67 fractions. Chrysin (**7**) directly precipitated in F28 without further processing. Four major components were isolated by semipreparative reversed phase HPLC after evaluation of all combined fractions by LC-MS. Partial separation on silica facilitated the consecutive isolation of the main bibenzyl **9** and stilbene **10** in *Pinus cembra* heartwood, as well as a mixture of both. When a combination of both substances was present in the same CC fraction (F20–22), they could not be isolated separately, as they eluted in one peak on RP18 phase at identical retention times. DHPSM (**9**) and PSM (**10**) proved to have interesting pharmacological potential in the past, showing anticancer and chemosensitizing effects of different potencies against human cancer cell lines [26,27]. Both substances also showed a wide spectrum of antibiotic and antifungal properties [11,28]. Two major flavonoids **8** and **12** could also be isolated. Results as confirmed by NMR (Supplementary Materials Figures S1–S4, Tables S3–S6) are given in Table 2.

### 2.3. Chromatographic Optimization for HPLC Separation of Isolated Compounds

Qualitative UHPLC analysis showed that bibenzyl **9** and its corresponding stilbene **10** were not distinguishable by their retention behavior on a Zorbax Eclipse Plus C18 column (EclC18) when eluting with a linear gradient of increasing concentrations of acetonitrile, as is common practice for HPLC analytical screenings of plant extracts (Figure 1). This poses an analytical problem that, to our knowledge, has not been tackled in literature before and could potentially lead to difficulties in detecting pairs of stilbenes and bibenzyls in the analysis of plant extracts via HPLC. The mixture of **9** and **10** isolated from F20–22 was therefore used for further analytical optimization by variation of stationary phase and mobile phase gradient.

Three different HPLC columns were selected for testing and optimizing separation of substances **9** and **10**: EclC18, that was also used for qualitative analysis of the ethanolic extract and fractions, a Kinetex C18 (KtxC18) and a Kinetex PFP (KtxPFP) of identical dimensions and core shell particle size, but with a pentafluorophenyl reversed stationary phase. The results are summarized in Table 3, with resolution values calculated from full width at half maximum (FWHM). A detection wavelength of 210 nm was chosen in accordance with strong UV absorption presented by both substances. Three-dimensional chromatograms (190–500 nm) and chromatograms at 210 nm are given in Supplementary Materials (Figures S5–S10).

As expected, gradient elution of **9** and **10** on EclC18 showed no separation. Gradient elution on KtxC18 led to similar results. However, a peak shoulder was visible at λ = 210 nm, which indicates slight separation. On KtxPFP, it was possible to separate **9** and **10** using gradient elution (R_S_ = 1.90). Isocratic elution on EclC18 and KtxC18 facilitated peak separation at 30% acetonitrile (R_S_ = 1.68, R_S_ = 1.57), although retention times were high, doubling from KtxC18 (24.787, 27.267 min) to EclC18 (51.243, 55.020 min). Elution on KtxPFP at 40% acetonitrile gave peak separation at remarkably low retention times (5.620, 6.237 min) with high resolution (R_S_ = 2.55). According to information by the manufacturer, a pentafluorophenyl stationary phase offers high degrees of steric interaction and can therefore improve the separation of structural isomers. Furthermore, fluorine groups with high electronegativity are present. It can be assumed that a combination of steric and polar interactions leads to the separation of compounds **9** and **10**, which only differ by one central double bond in the molecules.

### 2.4. Application to Extract Sample

As separation of isolated substances **9** and **10** had been achieved using gradient elution on KtxPFP, it was then tested on an additional *Pinus cembra* heartwood ethanolic extract and produced according to the method given under 3.3. Elution with the beforementioned linear gradient of 20 to 100% acetonitrile simulated a real-life sample in plant extract screening via HPLC and analyses were performed on all three columns (EclC18, KtxC18, KtxPFP), to work out potential differences.

All comparative results for the main bibenzyl and stilbene **9** and **10**, as well as retention of major flavonoids **7**, **8**, **11** and **12** at a detection wavelength of 210 nm are given in Table 4. Three-dimensional chromatograms (190–500 nm) and chromatograms at 210 nm can be found in Supplementary Materials (Figures S11–S13).

In the more complex matrix of the extract solution, separation of **9** and **10** by gradient elution was only successful on KtxPFP, comparable to results for isolated substances (R_S_ = 1.90). Additionally, effects on flavonoid separation became obvious, with **11** and **12** being separated (R_S_ = 1.25), which was also not possible on EclC18 during initial qualitative analysis. On KtxC18, which has identical column parameters to KtxPFP except for stationary phase type, **11** and **12,** differing by a double bond between C-2 and C-3, were also separated, but with lower resolution (R_S_ = 0.88). Slight separation of **9** and **10** within the same peak could be visualized in 3D chromatograms. However, out of all three columns, **7** and **8**, differing from each other by a double bond between C-2 and C-3, were only separated on EclC18 (R_S_ = 2.44), with no separation for **9** and **10** as well as **11** and **12**. EclC18 is a double-endcapped C18 stationary phase on a special porous silica support, which according to the manufacturer is designed to reduce strong absorption of basic and highly polar compounds. This could have improved the separation of **7** and **8**, having no methoxyl moiety in contrast to **9**–**12**, in comparison to other columns.

## 3. Materials and Methods

### 3.1. Plant Material

All the investigations were based on a complete radial cut disk of a *Pinus cembra* trunk, with approximate thickness of 4.5 cm and a diameter of 26.6 to 28.3 cm. The age of the tree was about 54 years. Material was obtained from the sawmill “Sägewerk und Hobelwerk Seebacher” (Himmelberg, Carinthia, Austria) in July 2021. A certificate of origin for the region of Nockberge, Austria, was given in written form. A voucher specimen was deposited at the Department of Pharmacognosy, University of Graz.

For analytical investigations, two sets of eight drillings through heartwood areas were made with a flat milling drill and wood chips combined to yield 45.79 g and 45.18 g of heartwood, respectively. Wood chips were freeze-dried using a VirTis BenchTop Pro freeze-drier (SP Industries, Warminster, PA, USA) for 24 h before grinding in a Retsch ZM100 centrifugal mill with 2.0 mm mesh (Retsch, Haan, Nordrhein-Westfalen, Germany).

### 3.2. Solvents and Reference Substances

Solvents for different steps in analyses were partially denatured ethanol (AustrAlco, Spillern, Austria) for extraction, hexane, ethyl acetate (both Carl Roth, Karlsruhe, Baden-Württemberg, Germany) and methanol (VWR, Radnor, PA, USA) for open column chromatography and TLC, chloroform-d_1_ (VWR) and pyridine-d_5_ (Armar, Döttingen, Aargau, Switzerland) for NMR.

HPLC mobile phases consisted of ultrapure water prepared from deionized water with a Barnstead MicroPure system (Thermo Fisher Scientific, Waltham, MA, USA), HPLC-grade acetonitrile (VWR) and formic acid for LC-MS (Honeywell, Charlotte, NC, USA).

Reference substances were abietic acid contributed by the Department of Pharmaceutical Chemistry, University of Graz, Austria, apigenin (Sigma-Aldrich, St. Louis, MO, USA), chrysin, pinocembrin-7-methyl ether (pinostrobin), linoleic acid (all Carl Roth) and oleic acid (Fluka, Buchs, St. Gallen, Switzerland).

### 3.3. Extraction and Fractionation

Two separate Soxhlet extractions of 40.95 g and 41.36 g of plant material were carried out with 500 mL of ethanol each for 24 h. The native extracts were evaporated to dryness on a rotary evaporator (Büchi R-100, Büchi, Flawil, St. Gallen, Switzerland). A total of 2.63 g of dry extract yielded from the first Soxhlet extraction were separated by open-column chromatography on 25 g of silica (40–63 µm, VWR) with hexane, ethyl acetate and methanol in solvent systems of rising polarity, leading to 67 fractions.

TLC analyses of fractions were carried out on Silica Gel 60 F_254_ aluminium plates (Merck, Darmstadt, Hessen, Germany) with mobile phase systems hexane/ethyl acetate/methanol = 75/20/5 (*v*/*v*) for apolar fractions and hexane/ethyl acetate/methanol = 60/20/20 (*v*/*v*) for polar fractions. The staining reagent consisted of 0.5 mL of 4-anisaldehyde, 10 mL of acetic acid (both Carl Roth), 85 mL of methanol and 5 mL of sulfuric acid (Carl Roth). After spraying, the plates were heated at 105 °C for 5 min.

### 3.4. UHPLC-DAD-ESI-MS^n^

Analyses of the first ethanolic extract, combined fractions and reference substances were conducted on a Dionex UltiMate 3000 RS system equipped with a DAD detector and coupled to an LTQ XL linear ion-trap mass spectrometer with ESI ion source (allThermo Fisher Scientific, Waltham, MA, USA). A Zorbax Eclipse Plus C18 column, 2.1 × 100 mm and 1.8 µm particle size (Agilent, Santa Clara, CA, USA) served as stationary phase, while the mobile phase was made up of water +0.1% formic acid (A) and acetonitrile (B). Gradient elution at a flow rate of 0.25 mL/min started at 20% B, rising to 100% B at 20.0 min, followed by a plateau of 100% B to 23.0 min and a drop back to 20% B at 23.5 min, which was kept stable until 29 min for re-equilibration. Column temperature was 35 °C. Injection volumes were 2 µL for dry ethanolic extract in methanol (5 mg/mL) and 1 µL for reference substances dissolved in methanol (1 mg/mL) as well as subfractions of varying concentrations.

DAD-UV spectra were recorded in a wavelength range of 190 to 700 nm. Mass spectral detection was performed in the range of *m*/*z* 50 to 2000 amu, with conditions set as follows: source heater temperature 250 °C, sheath gas flow 50 arb (arbitrary units), auxiliary gas flow 8 arb, source voltage 4.0 kV for ESI negative mode and 4.2 kV for ESI positive mode.

### 3.5. HPLC-DAD

A separate Dionex UltiMate 3000 RS system was used for analytical optimization without coupling to MS. Chromatographic conditions were the same as for qualitative UHPLC analysis, with the additional use of a Kinetex C18 column, 100 × 2.1 mm, 2.6 μm column and a Kinetex PFP column, 100 × 2.1 mm, 2.6 μm (both Phenomenex, Torrance, CA, USA). Samples of isolated compounds dissolved in methanol (1 mg/mL) were injected at 1 µL; a second dry ethanolic extract in methanol (5 mg/mL) had an injection volume of 2 µL.

### 3.6. Semipreparative HPLC

Semipreparative HPLC was carried out on a Shimadzu system consisting of DGU-20A5R degassing unit, LC-20AT solvent delivery pump, SIL-10AF autosampler, CBM-20A controller, CTO-20AC column oven, SPD-M20A diode array detector and FRC-10A fraction collector (all Shimadzu, Kyoto, Kinki, Japan). Stationary phase was a Luna C-10(2) column with 250 × 10 mm and 10 µm particle size (Phenomenex). Mobile phase consisted of water (A) and acetonitrile (B). Elution at 4 mL/min and 35 °C column temperature started at 40% B, rising to 100% B at 20.0 min. All compounds of interest eluted within this gradient, followed by varying plateaus of 100% B (0–5 min duration) for column cleaning and re-equilibration at 40% B. Injection volume was 200 µL.

### 3.7. NMR

Isolated substances **9** and **10** were measured on a Bruker NEO NMR-spectrometer (Bruker Corporation; Billerica, MA, USA) at 400 MHz in chloroform-d_1_. Substances **8** and **12** were measured on a Bruker Advance II NMR-spectrometer (Bruker Corporation) at 700 MHz in pyridine-d_5_. All measurements were performed with an internal standard of TMS. Recorded data sets were ^1^H and ^13^C, as well as 2D experiments COSY, HSQC and HMBC.

## 4. Conclusions

The present study investigating constituents of *Pinus cembra* heartwood with a focus on flavonoids, stilbenes and bibenzyls has shown early results for equivalent morphological plant material, generated in the 1940s to 1960s to be mostly true, yet expandable. We employed modern instrument-based HPLC techniques in combination with isolation and NMR spectroscopy to find substances **5** to **12** in alignment with these literature references, proving remarkable phytochemical analyses at the time [4,5,6].

However, we could not find evidence for the presence of pinobanksin, as described by Erdtman et al. in 1966 [6]. In addition, substances **1** to **4** and **13** have, to our knowledge, not yet been described for *Pinus cembra*.

This contribution to the knowledge of *Pinus cembra* heartwood phytochemistry also brings some clarity in consideration of reported variances in composition of different morphological parts of *Pinus cembra*. As for stilbenes and bibenzyls, Coniglio et al. find PS (**6**) and DHPS (**5**) in knotwood, while Patyra et al. report only PSM (**10**) in branch wood [15,16]. Both detect no DHPSM (**9**), while Erdtman et al., Lindstedt et al. and Willför et al. find it in knot- and heartwood [4,5,6,9]. For flavonoids, pinocembrin (**8**) aligns for knotwood and heartwood investigations, while other components vary as well.

In consecutive analyses, HPLC comparisons using different reversed phase materials as stationary phases showed a pentafluorophenyl reversed phase (KtxPFP) to be able to separate bibenzyl DHPSM (**9**) and stilbene PSM (**10**), which was not possible on two different C18 stationary phases (EclC18, KtxC18). Flavonoids pinostrobin (**11**) and tectochrysin (**12**) could be separated on KtxPFP and KtxC18. However, flavonoids chrysin (**7**) and pinocembrin (**8**) were only separated on EclC18.

Results show that a combination of different reversed phase columns could be recommended for HPLC screening of plant extracts using gradient elution, as good chromatographic resolution for several substances of interest is desired. This should especially be considered for UV detection, as overlays of coeluting substances make them hard to identify by UV-fingerprint, or when classifying substance groups through UV spectra. Quantification and isolation of individual components will also not be possible. In MS detection, coeluting substances can often be differentiated by their molecular ion when tracking specific *m*/*z* values using single ion monitoring. For screening purposes, good chromatographic resolution of substances should still be aimed for. Furthermore, chromatographic techniques coupled with mass spectrometry are destructive and therefore not useful for isolation of natural products. DHPSM (**9**) and PSM (**10**) proved to have interesting pharmacological potential in the past, showing anticancer and chemosensitizing effects of different potencies against human cancer cell lines [26,27]. Both substances also showed a wide spectrum of antibiotic and antifungal properties [11,28]. Still, repeated CC and HPLC were often necessary for isolation before biological testing. This could be improved by implementing HPLC method development employing different stationary phases in the fast and cost-effective isolation of these substances for further pharmacological testing.

## Figures and Tables

**Table 1:** Qualitative analysis of *Pinus cembra* heartwood ethanolic extract.

Nr.	Rt [min]	Mol. Mass	[M-H]^−^	[M+H]^+^	UV_max_ [nm]	Group	Substance
**1**	4.50	288	287	289	228, 291, 330 sh	Flavanonol	Aromadendrin *
**2**	6.82	270	269	271	268, 335	Flavone	Apigenin ^a^*
**3**	6.83	272	271	273	290, 330 sh	Flavanone	Naringenin *
**4**	7.84	242	241	243	217, 237 sh, 307, 318	Stilbene	Pinostilbene *
**5**	8.50	214	213	215	201, 276	Bibenzyl	Dihydropinosylvin [5,6,16]
**6**	8.56	212	211	213	210, 228 sh, 300, 308	Stilbene	Pinosylvin [5,6,16]
**7**	9.60	254	253	255	269, 313	Flavone	Chrysin ^a^ [4,5,6,16]
**8**	9.91	256	255	257	290, 330 sh	Flavanone	Pinocembrin ^b^ [4,5,6,10,11,16]
**9**	11.36	228	227	229	205, 276	Bibenzyl	Dihydropinosylvin monomethyl ether ^b^ [5,9]
**10**	11.36	226	225	227	211, 227 sh, 300, 308	Stilbene	Pinosylvin monomethyl ether ^b^ [4,5,9,15]
**11**	13.05	270	269	271	289, 330 sh	Flavanone	Pinostrobin ^a^ [5,6]
**12**	13.05	268	267	269	268, 310	Flavone	Tectochrysin ^b^ [4,5,6]
**13**	14.77	240	- ^1^	241	206, 229 sh, 300, 307	Stilbene	Pinosylvin dimethyl ether *
**14**	18.24	278	277	279	193	Fatty acid	Linolenic acid [13]
**15**	18.36	302	301	303	243	Diterpenic acid	Abietic acid ^a^ [13,15]
**16**	19.29	280	279	-	194	Fatty acid	Linoleic acid ^a^ [13]
**17**	20.78	282	281	-	195	Fatty acid	Oleic acid ^a^ [13]

^a^ Confirmed via comparison with reference standard; ^b^ confirmed by NMR after isolation; * first report for *Pinus cembra*; ^1^ no detection due to weak ionization in respective mode.

**Table 2:** Substances isolated from CC fractions of *Pinus cembra* heartwood ethanolic extract by semi-preparative HPLC on RP18 stationary phase. Identification was confirmed by NMR analysis.

CC Fraction	Isolated Substance (Quantity in mg)
F17–18	**9** (1.5)
F19	**9** (49.3)
F20–22	**9** + **10 ^1^** (22.9), **12** (2.9)
F23–25	**8** (7.6), **10** (12.2), **12** (1.1)
F28	**7** (9.5)

^1^ Mixture of both substances with identical retention times.

**Table 3:** HPLC studies on mixture of isolated compounds **9** and **10**, UV-detection at λ = 210 nm.

HPLC Column	Mobile Phase B ^1^	Substance	Rt [min]	Rs ^2^
EclC18	20–100%	**9** + **10**	11.460	- ^3^
	30%	**9**	51.243	
		**10**	55.020	1.68
KtxC18	20–100%	**9** + **10**	9.893	-
	30%	**9**	24.787	
		**10**	27.267	1.57
KtxPFP	20–100%	**9**	9.337	
		**10**	9.607	1.90
	40%	**9**	5.620	
		**10**	6.237	2.55

^1^ HPLC-grade acetonitrile; ^2^ resolution (FWHM); ^3^ coelution of substances.

**Table 4:** HPLC screening of *Pinus cembra* heartwood ethanolic extract on different columns using gradient elution, UV-detection at λ = 210 nm.

HPLC Column	Substance	Rt [min]	Rs ^1^
EclC18	**7**	9.673	
	**8**	9.980	2.44
	**9 + 10**	11.433	- ^2^
	**11 + 12**	13.127	-
KtxC18	**7 + 8**	8.567	-
	**9 + 10**	9.867	-
	**11**	11.303	
	**12**	11.487	0.88
KtxPFP	**7 + 8**	8.480	-
	**9**	9.317	
	**10**	9.593	1.90
	**11**	10.810	
	**12**	10.967	1.25

^1^ Resolution (FWHM); ^2^ coelution of substances.

## Data Availability

Data are included in this article.

## Supplementary Materials

The following supporting information can be downloaded at: https://www.mdpi.com/article/10.3390/plants12193388/s1, Figure S1: Structure of substance **9** Dihydropinosylvin monomethyl ether; Figure S2: Structure of substance **10** Pinosylvin monomethyl ether; Figure S3: Structure of substance **12** Tectochrysin; Figure S4: Structure of substance **8** Pinocembrin; Figure S5: HPLC chromatogram (5–15 min) of a mixture of **9** and **10**, gradient elution (20–100% B), column EclC18; A: 210 nm, B: 3D field (190–500 nm); Figure S6: HPLC chromatogram of a mixture of **9** and **10**, isocratic elution (30% B), column EclC18; A: 210 nm, B: 3D field (190–500 nm); Figure S7: HPLC chromatogram (5–15 min) of a mixture of **9** and **10**, gradient elution (20–100% B), column KtxC18; A: 210 nm, B: 3D field (190–500 nm); Figure S8: HPLC chromatogram of a mixture of **9** and **10**, isocratic elution (30% B), column KtxC18; A: 210 nm, B: 3D field (190–500 nm); Figure S9: HPLC chromatogram (5–15 min) of a mixture of **9** and **10**, gradient elution (20–100% B), column KtxPFP; A: 210 nm, B: 3D field (190–500 nm); Figure S10: HPLC chromatogram of a mixture of **9** and **10**, isocratic elution (40% B), column KtxPFP; A: 210 nm, B: 3D field (190–500 nm); Figure S11: HPLC chromatogram (8–18 min) of a *Pinus cembra* heartwood ethanolic extract, gradient elution (20–100% B), column EclC18; A: 210 nm, B: 3D field (190–500 nm); Figure S12: HPLC chromatogram (8–18 min) of a *Pinus cembra* heartwood ethanolic extract, gradient elution (20–100% B), column KtxC18; A: 210 nm, B: 3D field (190–500 nm); Figure S13: HPLC chromatogram (5–15 min) of a *Pinus cembra* heartwood ethanolic extract, gradient elution (20–100% B), column KtxPFP; A: 210 nm, B: 3D field (190–500 nm); Table S1: Qualitative analysis of *Pinus cembra* heartwood ethanolic extract. MS^n^ data in ESI positive mode; Table S2: Qualitative analysis of *Pinus cembra* heartwood ethanolic extract. MS^n^ data in ESI negative mode; Table S3: NMR-data of substance **9** Dihydropinosylvin monomethyl ether in CDCl_3_ (400 MHz); Table S4: NMR-data of substance **10** Pinosylvin monomethyl ether in CDCl_3_ (400 MHz); Table S5: NMR-data of substance **12** Tectochrysin in pyridin-d_5_ (700 MHz); Table S6: NMR-data of substance **8** Pinocembrin in pyridin-d_5_ (700 MHz).
