# Supplementary material for: Phytochemical Analysis of Pinus cembra Heartwood—UHPLC-DAD-ESI-MSn with Focus on Flavonoids, Stilbenes, Bibenzyls and Improved HPLC Separation
Source: Plants (Basel). 2023 Sep 25;12(19):3388. doi: 10.3390/plants12193388 (PMC10574252; doi:10.3390/plants12193388)
Supplement: Supplementary file 1 [file plants-12-03388-s001.zip › plants-2580681-supplementary.pdf]

Article

Phytochemical Analysis of *Pinus cembra* Heartwood – UHPLC-DAD-ESI-MS^n with Focus on Flavonoids, Stilbenes, Bibenzyls and Improved HPLC Separation

Fabian Alperth [1], Anna Schneebauer [1], Olaf Kunert [2] and Franz Bucar [1,*]

[1] Institute of Pharmaceutical Sciences, Department of Pharmacognosy, University of Graz, Beethovenstraße 8, 8010 Graz, Austria; fabian.alperth@uni-graz.at; schneebauer.anna@gmail.com;
[2] Institute of Pharmaceutical Sciences, Department of Pharmaceutical Chemistry, University of Graz, Universitätsplatz 1, 8010 Graz, Austria; olaf.kunert@uni-graz.at
* Correspondence: franz.bucar@uni-graz.at; Tel.: +43-316-380-5531

Supplementary Materials

MS^n results in ESI positive and negative mode are given in Tables S1 and S2, with threshold for listed fragment ions in MS^n set as ⩾ 10% relative intensity.

Table S1. Qualitative analysis of *Pinus cembra* heartwood ethanolic extract. MS^n data in ESI positive mode.

Nr.	RT [min]	Molecular Ion [m/z]	MS^n [m/z], Relative Intensity (%)	Molecular Weight [g/mol]	Substance
**1**	4.50	289 [M+H]$^+$	MS^2[289]: 271 (100), 243 (51), 195 (12), 153 (64)<br>MS^3[271]: 243 (100)<br>MS^4[243]: 215 (100), 149 (86)	288	Aromadendrin
**2**	6.82	271 [M+H]$^+$	MS^2[271]: 272 (10), 271 (100), 229 (26), 225 (14), 153 (81), 145 (11)<br>MS^3[271]: 271 (100), 229 (24), 153 (64)	270	Apigenin
**3**	6.83	273 [M+H]$^+$	MS^2[273]: 153 (100), 147 (85)<br>MS^3[153]: 153 (100), 111 (23), 67 (42)<br>MS^4[153]: 153 (100), 109 (19), 67 (28)	272	Naringenin
**4**	7.84	243 [M+H]$^+$	MS^2[243]: 225 (35), 211 (16), 197 (11), 159 (12), 149 (100), 147 (20), 145 (12), 133 (35), 121 (19), 119 (17)<br>MS^3[149]: 121 (100)<br>MS^4[121]: 121 (100), 93 (21)	242	Pinostilbene
**5**	8.50	215 [M+H]$^+$	MS^2[215]: 137 (95), 105 (100)<br>MS^3[105]: 105 (100)<br>MS^4[105]: 105 (100)	214	Dihydropinosylvin
**6**	8.56	213 [M+H]$^+$	MS^2[213]: 135 (100)<br>MS^3[135]: 135 (10), 107 (100)<br>MS^4[107]: 107 (100)	212	Pinosylvin
**7**	9.60	255 [M+H]$^+$	MS^2[255]: 256 (13), 255 (100), 213 (37), 209 (23), 187 (15), 153 (73), 129 (10)<br>MS^3[255]: 255 (100), 214 (13), 213 (42), 209 (16) 153 (59)	254	Chrysin
**8**	9.91	257 [M+H]$^+$	MS^2[257]: 215 (23), 179 (10), 173 (27), 153 (100), 131 (83)<br>MS^3[153]: 153 (100), 111 (35), 109 (10), 67 (47)	256	Pinocembrin
**9**	11.36	229 [M+H]$^+$	MS^2[229]: 151 (87), 137 (13), 105 (100)<br>MS^3[105]: 105 (100)<br>MS^4[105]: 105 (100)	228	Dihydropinosylvin monomethyl ether

**10**	11.36	227 [M+H]+	MS2[227]: 209 (14), 195 (13) 181 (16), 149 (100), 143 (30), 141 (12), 135 (17) 131 (34), 129 (24), 121 (12), 117 (45)<br>MS3[149]: 121 (100)<br>MS4[121]: 121 (100), 93 (15)	226	Pinosylvin monomethyl ether
**11**	13.05	271 [M+H]+	MS2[271]: 173 (16), 167 (100), 131 (75)<br>MS3[167]: 167 (100), 127 (25), 123 (25), 67 (47)<br>MS4[167]: 167 (100), 127 (17), 123 (15), 67 (14)	270	Pinostrobin
**12**	13.05	269 [M+H]+	MS2[269]: 269 (43), 255 (19), 254 (100), 226 (20), 209 (18), 167 (21)<br>MS3[254]: 226 (100)<br>MS4[226]: 226 (10), 208 (49), 198 (66), 197 (100), 181 (96), 180 (37) 171 (97), 170 (67), 169 (26), 152 (15), 144 (13) 124 (17)	268	Tectochrysin
**13**	14.77	241 [M+H]+	MS2[241]: 223 (17), 213 (50), 209 (60), 181 (59), 149 (22), 143 (46), 141 (23), 137 (11), 129 (70), 117 (100), 113 (44) 111 (84), 91 (10)<br>MS3[117]: 117 (100)<br>MS4[117]: 117 (100)	240	Pinosylvin dimethyl ether
**14**	18.24	279 [M+H]+	MS2[279]: 261 (100), 243 (36), 195 (10), 181 (11)<br>MS3[261]: 243 (100)<br>MS4[243]: 215 (34), 201 (45), 187 (88), 175 (11) 173 (100), 161 (37), 159 (52), 149 (11), 147 (25), 145 (38), 135 (16), 133 (17), 131 (34), 121 (18), 119 (10)	278	Linolenic acid
**15**	18.36	303 [M+H]+	MS2[303]: 285 (47), 257 (100), 123 (56)<br>MS3[257]: 229 (20), 215 (28), 201 (100), 187 (45), 175 (27), 173 (32), 163 (11), 161 (21), 159 (23), 149 (24), 147 (25), 145 (10), 135 (25), 133 (13), 123 (10), 109 (16)<br>MS4[201]: 173 (21), 159 (54), 145 (100), 131 (21), 119 (18), 107 (10), 105 (10)	302	Abietic acid
**16**	19.29	- [1]	-	280	Linoleic acid
**17**	20.78	-	-	282	Oleic acid

[1] No detection and/or fragmentation due to weak ionization in respective mode.

Table S2. Qualitative analysis of *Pinus cembra* heartwood ethanolic extract. MS^n data in ESI negative mode.

Nr.	RT [min]	Molecular Ion [m/z]	MS^n [m/z], Relative Intensity (%)	Molecular Weight [g/mol]	Substance
**1**	4.50	287 [M-H]-	MS2[287]: 259 (100), 243 (11)<br>MS3[259]: 241 (17), 215 (100), 173 (23), 172 (11), 165 (10), 151 (14), 125 (53)<br>MS4[215]: 200 (18), 173 (100), 172 (41), 158 (10)	288	Aromadendrin
**2**	6.82	269 [M-H]-	MS2[269]: 269 (15), 227 (15), 226 (12), 225 (100), 201 (25), 183 (15), 181 (11), 151 (18), 149 (36)<br>MS3[225]: 197 (40), 196 (20), 183 (38), 181 (100), 169 (14)<br>MS4[181]: 181 (100), 153 (25), 152 (30), 141 (11), 117 (15)	270	Apigenin
**3**	6.83	271 [M-H]-	MS2[271]: 177 (21), 151 (100)<br>MS3[151]: 107 (100)<br>MS4[107]: 107 (53), 65 (100)	272	Naringenin
**4**	7.84	241 [M-H]-	MS2[241]: 226 (100), 225 (97), 223 (10), 209 (12), 165 (17)<br>MS3[226]: 211 (15), 198 (100), 197 (47), 183 (29), 182 (24), 181 (52)	242	Pinostilbene

**5**	8.50	213 [M-H]-	- [1]	214	Dihydropinosylvin
**6**	8.56	211 [M-H]-	MS²[211]: 169 (100), 167 (75), 165 (12)<br>MS³[169]: 169 (100), 141 (70), 127 (26)	212	Pinosylvin
**7**	9.60	253 [M-H]-	MS²[253]: 253 (36), 211 (12), 210 (12), 209 (100), 181 (15), 180 (13)<br>MS³[209]: 209 (10), 181 (100), 180 (84), 167 (13), 165 (42), 153 (19)<br>MS⁴[181]: 181 (12), 153 (100), 139 (68)	254	Chrysin
**8**	9.91	255 [M-H]-	MS²[255]: 213 (100), 211 (42), 187 (16), 151 (28)<br>MS³[213]: 185 (100), 169 (26), 145 (16)<br>MS⁴[185]: 185 (100), 157 (20), 143 (80), 141 (65), 129 (16), 117 (12)	256	Pinocembrin
**9**	11.36	227 [M-H]-	-	228	Dihydropinosylvin monomethyl ether
**10**	11.36	225 [M-H]-	-	226	Pinosylvin monomethyl ether
**11**	13.05	269 [M-H]-	MS²[269]: 254 (100), 251 (60), 236 (30), 226 (17), 225 (12), 165 (29)<br>MS³[254]: 226 (100), 225 (71), 177 (16), 163 (13)<br>MS⁴[226]: 198 (100), 197 (14), 183 (10), 181 (14), 122 (82)	270	Pinostrobin
**12**	13.05	267 [M-H]-	-	268	Tectochrysin
**13**	14.77	-	-	240	Pinosylvin dimethyl ether
**14**	18.24	277 [M-H]-	MS²[277]: 259 (22), 233 (100)<br>MS³[233]: 191 (100), 179 (19), 177 (57)	278	Linolenic acid
**15**	18.36	301 [M-H]-	MS²[301]: 301 (39), 286 (53), 283 (40), 273 (26), 257 (75), 255 (100), 233 (10), 200 (14), 89 (11)	302	Abietic acid
**16**	19.29	279 [M-H]-	MS²[279]: 261 (100)<br>MS³[261]: 259 (63), 243 (100), 233 (19), 219 (19), 165 (11), 95 (12), 83 (30)	280	Linoleic acid
**17**	20.78	281 [M-H]-	MS²[281]: 263 (100)<br>MS³[263]: 245 (100), 235 (52), 233 (13), 221 (20), 195 (12), 193 (14), 183 (25), 179 (22), 167 (14), 155 (31), 153 (14), 137 (26), 127 (14) 125 (19), 123 (12), 111 (12), 97 (45), 95 (23), 83 (57), 81 (13)	282	Oleic acid

[1] No detection and/or fragmentation due to weak ionization in respective mode.

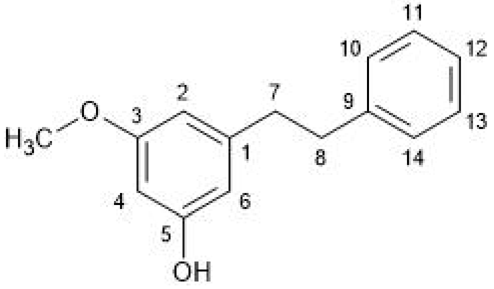

Figure S1. Structure of substance **9** Dihydropinosylvin monomethyl ether.

Table S3. NMR-data of substance **9** Dihydropinosylvin monomethyl ether in $CDCl_3$ (400 MHz).

Position	$\delta^{13}C$ [ppm], Type	$\delta^{1}H$ [ppm], Mult.[1] (J in Hz)
1	144.6, C	-
2	106.8, CH	6.32, t (2.0)
3	160.7, C	-
3-OCH_3	55.3, CH_3	3.72, s
4	99.1, CH	6.25, m
5	156.5, C	-
6	108.1, CH	6.25, m
7	37.9, CH_2	2.79 / 2.81, m
8	37.5, CH_2	2.86 / 2.88, m
9	141.6, C	-
10	128.4, CH	7.16, m
11	128.4, CH	7.27, t (7.5)
12	126.0, CH	7.18, m
13	128.4, CH	7.27, t (7.5)
14	128.4, CH	7.16, m

[1] s: singlet, d: doublet, t: triplet, m: multiplet.

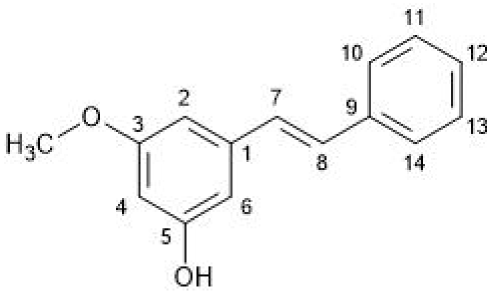

Figure S2. Structure of substance **10** Pinosylvin monomethyl ether.

Table S4. NMR-data of substance **10** Pinosylvin monomethyl ether in $CDCl_3$ (400 MHz).

Position	$\delta^{13}C$ [ppm], Type	$\delta^{1}H$ [ppm], Mult.[1] (J in Hz)
1	139.7, C	-
2	105.0, CH	6.65, t (1.5)
3	161.1, C	-
3-OCH_3	55.4, CH_3	3.82, s
4	101.0, CH	6.34, t (2.0)
5	156.8, C	-
6	105.9, CH	6.60, t (1.5)
7	128.3, CH	7.01, d (16.2)
8	129.4, CH	7.04, d (16.2)
9	137.0, C	-
10	126.6, CH	7.49, d (7.5)
11	128.7, CH	7.35, t (7.5)
12	127.8, CH	7.26, t (7.5)
13	128.7, CH	7.35, t (7.5)
14	126.6, CH	7.49, d (7.5)

[1] s: singlet, d: doublet, t: triplet.

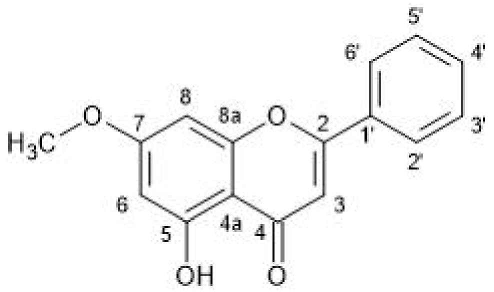

Figure S3. Structure of substance **12** Tectochrysin.

Table S5. NMR-data of substance **12** Tectochrysin in pyridin-d_5 (700 MHz).

Position	δ^{13}C [ppm], Type	δ^{1}H [ppm], Mult.[1] (J in Hz)
2	164.1, C	-
3	106.3, CH	7.02, s
4	182.9, C	-
4a	106.1, C	-
5	162.7, C	-
6	98.9, CH	6.63, brs
7	166.2, C	-
7-OCH_3	56.0, CH_3	3.79, s
8	93.0, CH	6.73, br
8a	158.2, C	-
1′	131.7, C	-
2′	126.8, CH	7.95, d (7.6)
3′	129.4, CH	7.49, m
4′	132.2, CH	7.51, m
5′	129.4, CH	7.49, m
6′	126.8, CH	7.95, d (7.6)

[1] s: singlet, brs: broad singlet, d: doublet, m: multiplet, br: broad signal.

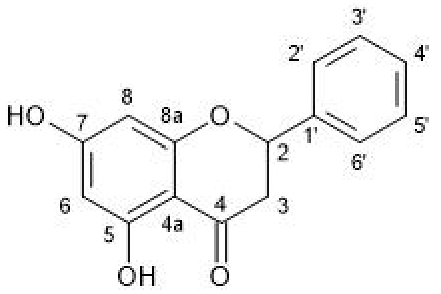

Figure S4. Structure of substance **8** Pinocembrin.

Table S6. NMR-data of substance **8** Pinocembrin in pyridin-d_5 (700 MHz).

Position	δ^{13}C [ppm], Type	δ[1]H [ppm], Mult.[1] (J in Hz)
2	79.6, CH	5.52, dd (12.8, 2.9)
3	43.4, CH_2	3.21 dd (16.8, 12.8) /
		2.90 dd (16.8, 2.9)
4	196.0, C	-
4a	102.9, C	-
5	165.2, C	-
6	97.4, CH	6.49, brs
7	168.7, C	-
8	96.2, CH	6.41, brs
8a	163.8, C	-
1′	139.5, C	-
2′	126.9, CH	7.58, d (7.8)
3′	129.1, CH	7.41, t (7.6)
4′	129.0, CH	7.36, t (7.4)
5′	129.1, CH	7.41, t (7.6)
6′	126.9, CH	7.58, d (7.8)

[1] brs: broad singlet, d: doublet, dd: doublet of doublets, t: triplet.

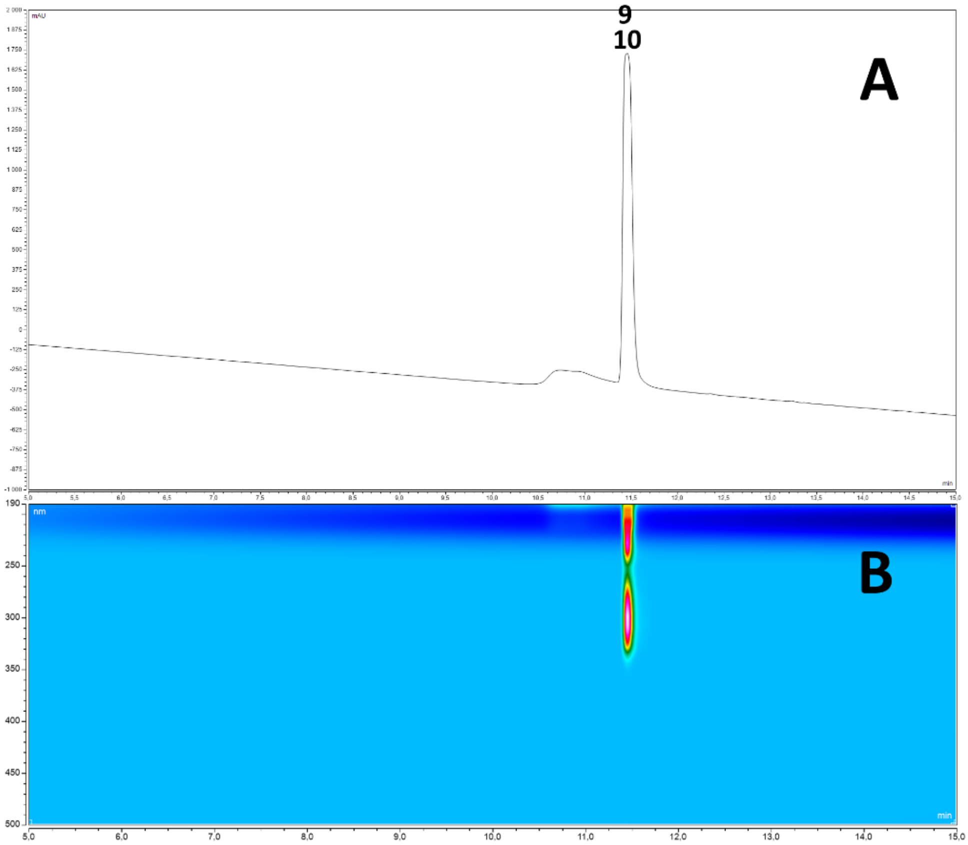

Figure S5. HPLC chromatogram (5-15 min) of a mixture of **9** and **10**, gradient elution (20-100 % B), column EclC18; A: 210 nm, B: 3D field (190-500 nm).

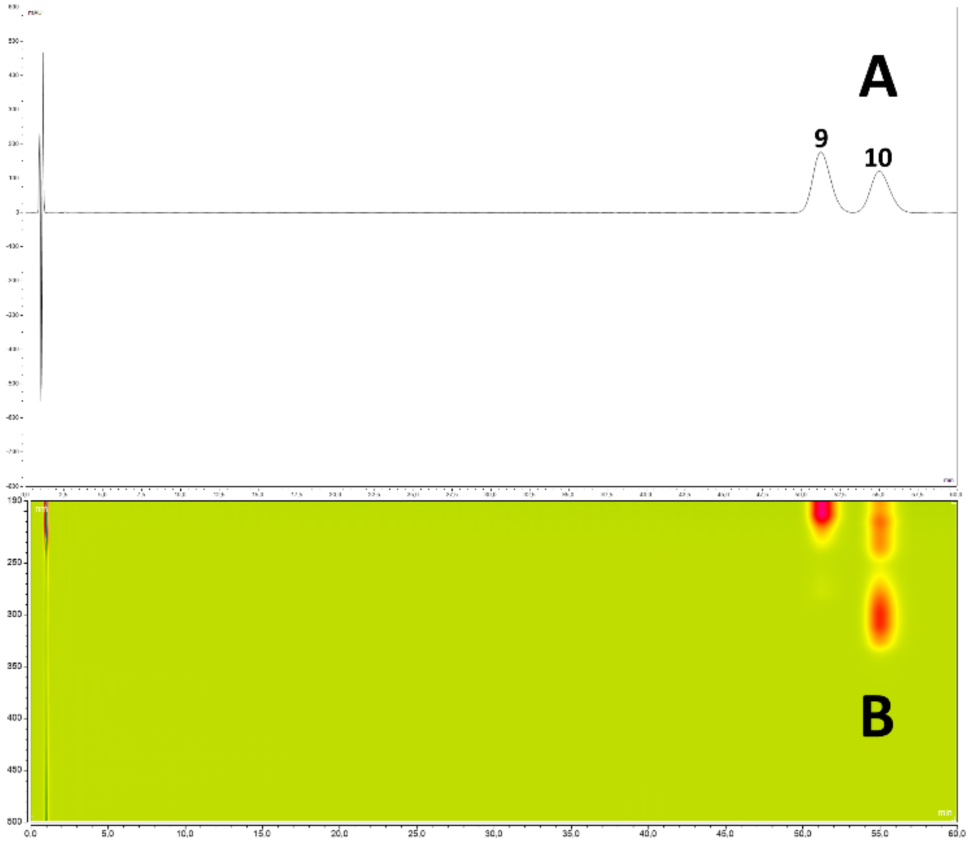

Figure S6. HPLC chromatogram of a mixture of **9** and **10**, isocratic elution (30 % B), column EclC18; A: 210 nm, B: 3D field (190-500 nm).

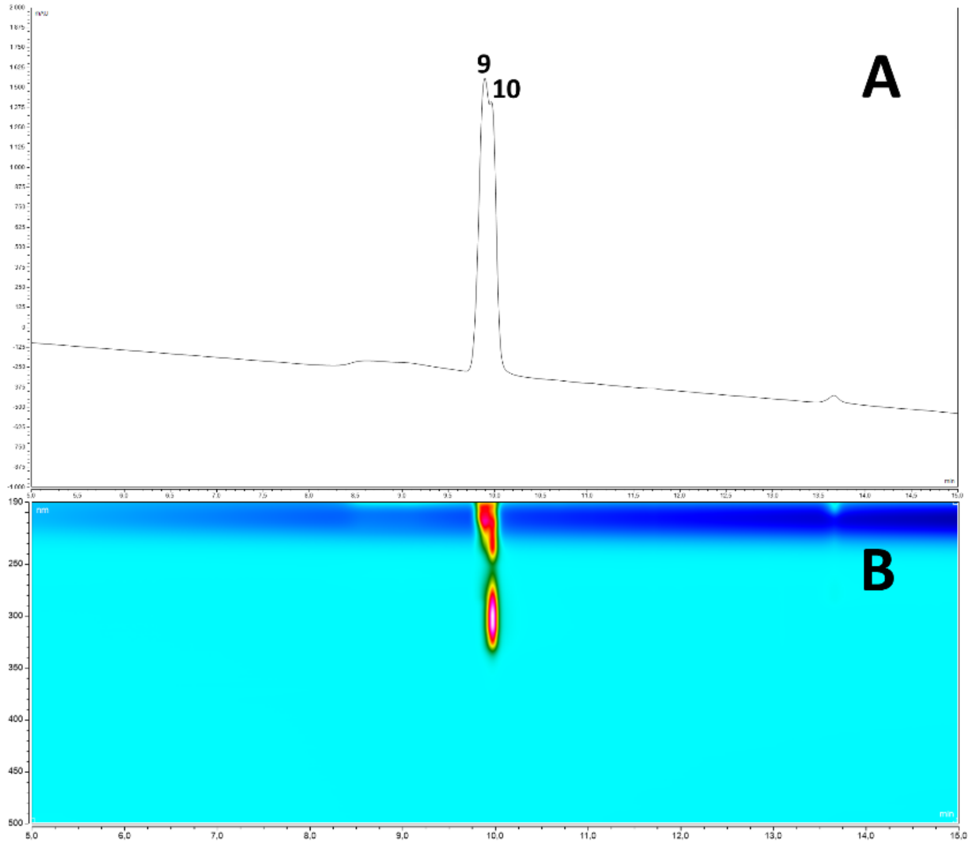

Figure S7. HPLC chromatogram (5-15 min) of a mixture of **9** and **10**, gradient elution (20-100 % B), column KtxC18; A: 210 nm, B: 3D field (190-500 nm).

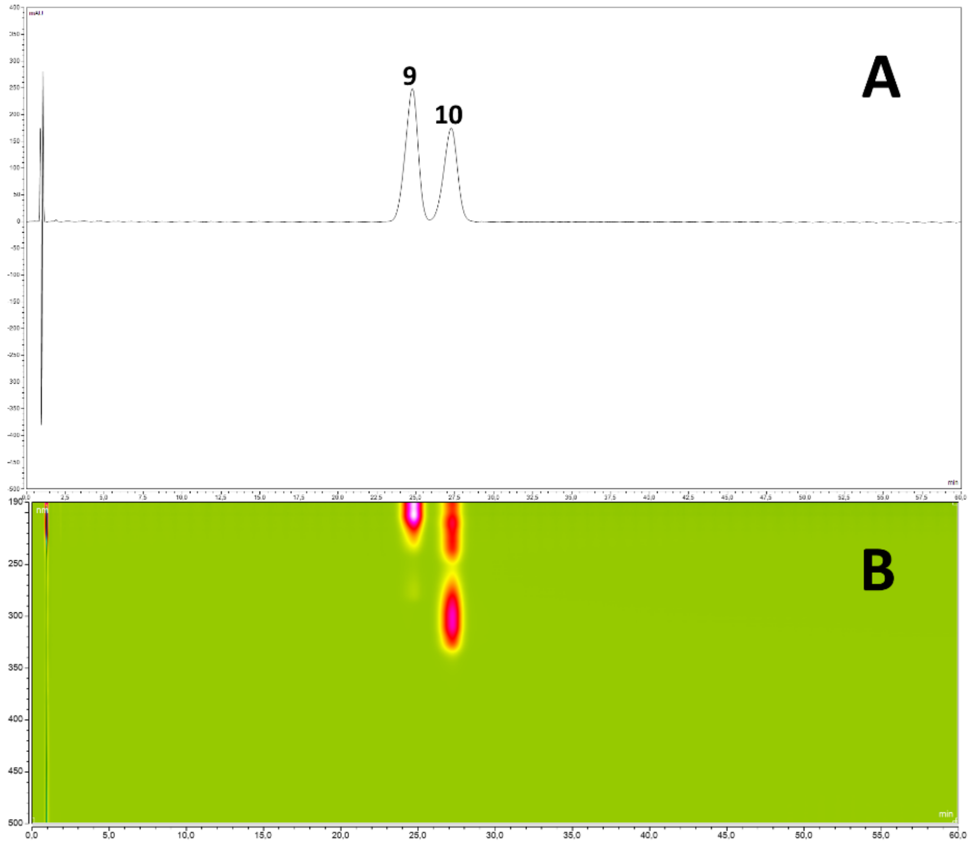

Figure S8. HPLC chromatogram of a mixture of **9** and **10**, isocratic elution (30 % B), column KtxC18; A: 210 nm, B: 3D field (190-500 nm).

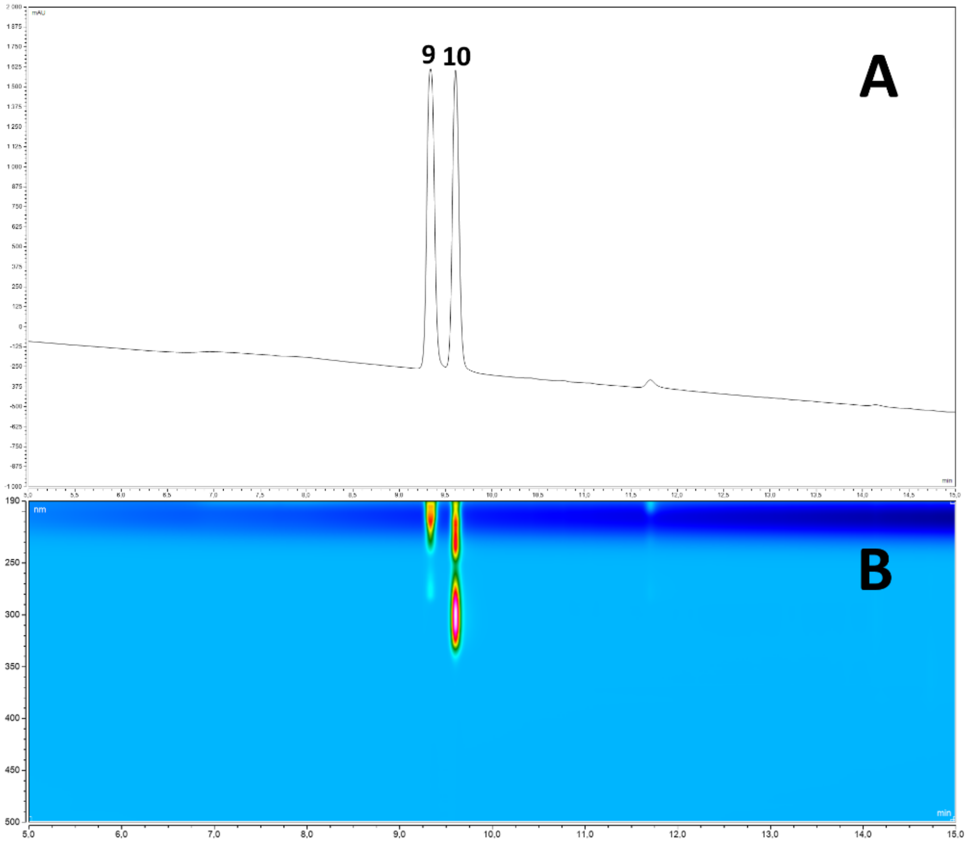

Figure S9. HPLC chromatogram (5-15 min) of a mixture of **9** and **10**, gradient elution (20-100 % B), column KtxPFP; A: 210 nm, B: 3D field (190-500 nm).

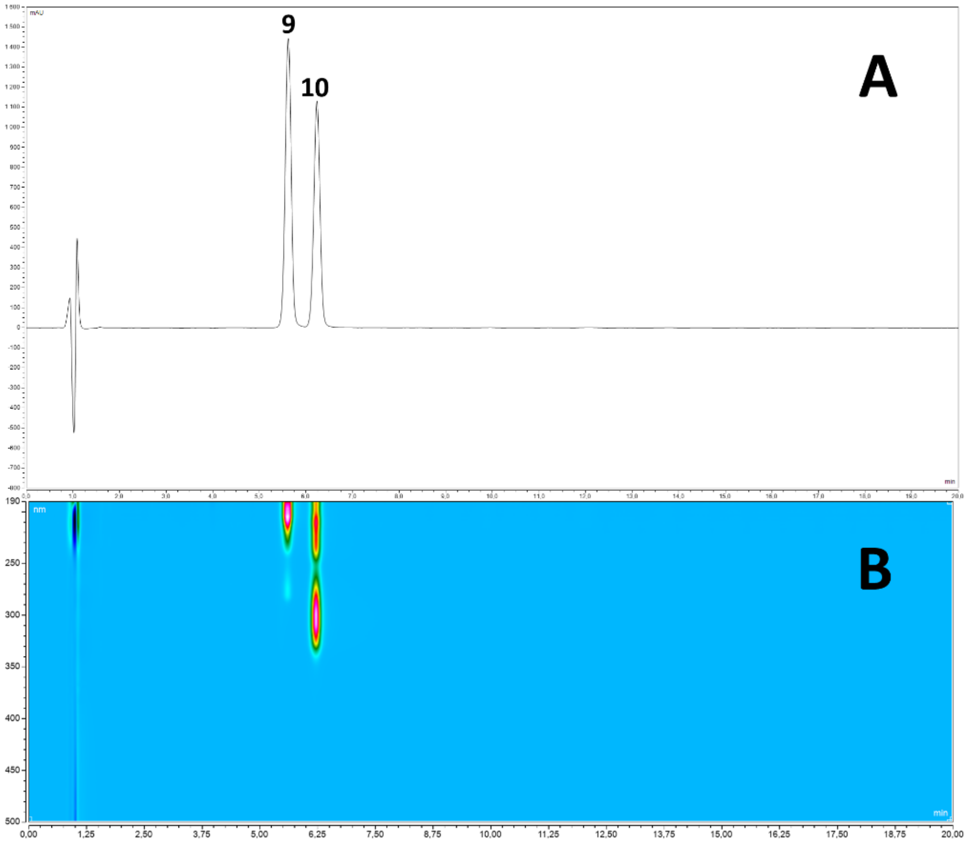

Figure S10. HPLC chromatogram of a mixture of **9** and **10**, isocratic elution (40 % B), column KtxPFP; A: 210 nm, B: 3D field (190-500 nm).

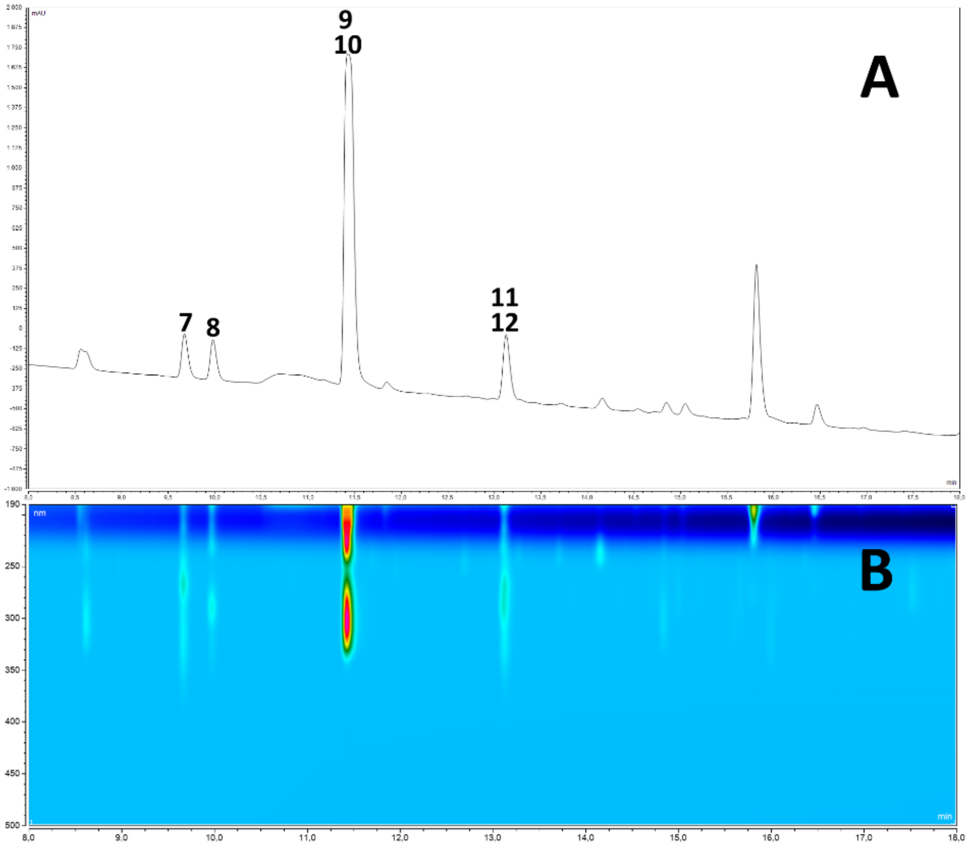

Figure S11. HPLC chromatogram (8-18 min) of a *Pinus cembra* heartwood ethanolic extract, gradient elution (20-100 % B), column EclC18; A: 210 nm, B: 3D field (190-500 nm).

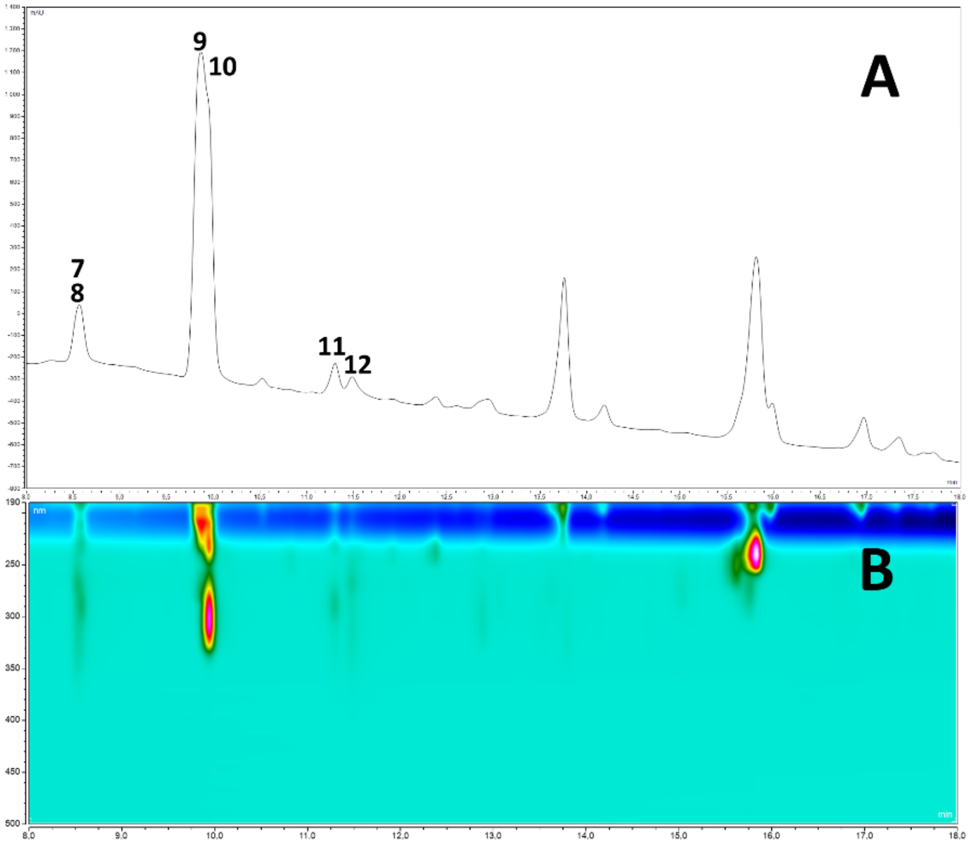

Figure S12. HPLC chromatogram (8-18 min) of a *Pinus cembra* heartwood ethanolic extract, gradient elution (20-100 % B), column KtxC18; A: 210 nm, B: 3D field (190-500 nm).

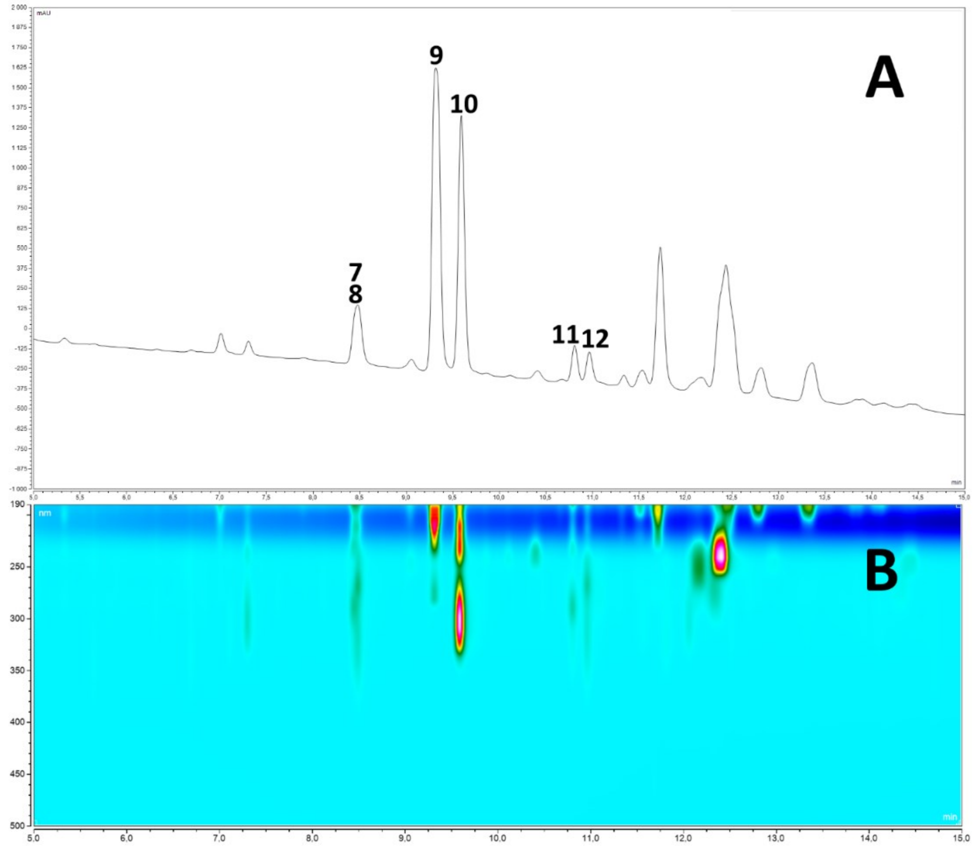

Figure S13. HPLC chromatogram (5-15 min) of a *Pinus cembra* heartwood ethanolic extract, gradient elution (20-100 % B), column KtxPFP; A: 210 nm, B: 3D field (190-500 nm).
